# Effect of Synaptic Transmission on Viral Fitness in HIV Infection

**DOI:** 10.1371/journal.pone.0048361

**Published:** 2012-11-15

**Authors:** Natalia L. Komarova, David N. Levy, Dominik Wodarz

**Affiliations:** 1 Department of Mathematics, University of California Irvine, Irvine, California, United States of America; 2 Department of Ecology and Evolution, University of California Irvine, Irvine, California, United States of America; 3 Department of Basic Science, New York University College of Dentistry, New York, New York, United States of America; Albert Einstein College of Medicine, United States of America

## Abstract

HIV can spread through its target cell population either via cell-free transmission, or by cell-to-cell transmission, presumably through virological synapses. Synaptic transmission entails the transfer of tens to hundreds of viruses per synapse, a fraction of which successfully integrate into the target cell genome. It is currently not understood how synaptic transmission affects viral fitness. Using a mathematical model, we investigate how different synaptic transmission strategies, defined by the number of viruses passed per synapse, influence the basic reproductive ratio of the virus, R_0_, and virus load. In the most basic scenario, the model suggests that R_0_ is maximized if a single virus particle is transferred per synapse. R_0_ decreases and the infection eventually cannot be maintained for larger numbers of transferred viruses, because multiple infection of the same cell wastes viruses that could otherwise enter uninfected cells. To explain the relatively large number of HIV copies transferred per synapse, we consider additional biological assumptions under which an intermediate number of viruses transferred per synapse could maximize R_0_. These include an increased burst size in multiply infected cells, the saturation of anti-viral factors upon infection of cells, and rate limiting steps during the process of synapse formation.

## Introduction

Human immunodeficiency virus (HIV) infection is characterized by a complex dynamic interplay between virus replication and specific immune responses, which eventually results in the development of AIDS. The rate of viral spread through the target cell population has been shown to influence the level of virus control and the pattern of disease progression [1], [2], [3]. Viral spread through the population of target cells can occur via two basic mechanisms [4], [5], [6], [7], [8], [9], [10]. (i) In cell-free spread, viruses are released from cells into the extracellular environment and infect susceptible targets that are encountered. (ii) In cell-cell spread, viruses can pass directly from one cell to another without entering the extracellular environment, presumably through the formation of virological synapses. On a per cell basis, cell to cell spread has been shown to be very effective [4]. Tens to hundreds of virus particles are transferred through synapses, a certain fraction of which successfully integrates into the genome of the target cell. This has been thought to confer an advantage to the virus population in a variety of settings [4], [11]. Synaptic transmission in HIV infection is considered to be particularly important in tissue sites, such as lymph nodes and the spleen, where cells have a relatively high likelihood to come into contact with each other and to form synapses. This can lead to the frequent multiple infection of target cells. Indeed, infected cells derived from the spleen of HIV-infected patients show an average of 3–4 viruses per cell [12], and synapse formation has been shown *in vitro* to lead to the co-transmission of multiple copies of HIV-1 across a single synapse [13]. This is in contrast to cell-free transmission, which typically leads to the transmission of single viral copies to target cells. Indeed, in the blood where cells mix more readily and synapse formation is less likely to occur, most infected cells have been found to contain a single copy of HIV-1 [14].

The occurrence of synaptic transmission in HIV infection brings up an evolutionary question. What is the optimal number of viruses transferred from a source cell to a target cell such that the rate of viral spread is maximized? Along similar lines, how does this optimum depend on the biological assumptions? These questions are investigated here with a new mathematical model that takes into account synaptic transmission of the virus. We vary the average number of viruses transferred through a synapse and investigate how this affects the basic reproductive ratio of the virus, a measure which quantifies how fast the virus spreads through its population of target cells. The basic reproductive ratio of the virus is shown to correlate with viral fitness. For simplicity, we will refer to the number of viruses transferred per synapse as the “viral strategy” in the rest of this paper.

In the simplest setting, the model gives rise to the surprising result that the optimal viral strategy is to transfer a single virus particle per synapse. Increasing the number of viruses transferred per synapse leads to a reduced basic reproductive ratio and to extinction of the infection. We subsequently examine conditions that could account for the emergence of synaptic transmission strategies that transfer of the order of 10^2^ viruses per synapse, typically observed in HIV infection [4], [6]. These include enhanced virus production in multiply infected cells, saturation of cellular anti-viral factors upon infections, and rate-limiting steps in the process of synapse formation. The relevance of these different mechanisms for HIV infection is discussed.

## Results

### The Model

#### Virus dynamics

**Figure 1:**
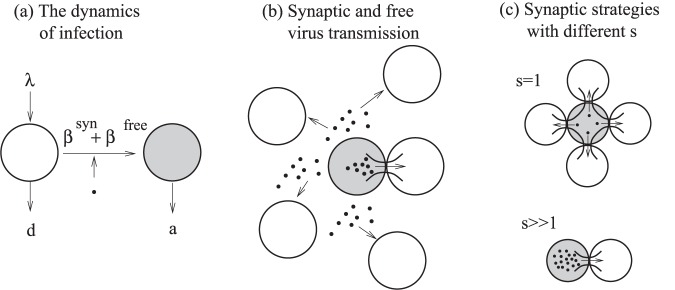
A schematic explaining the structure of the model. Here, uninfected cells are represented by white circles, infected cell by shaded circles, and viruses by black dots. (a) The overall virus dynamics, including production and death of target cells, the death of infected cells, and infection. (b) The process of infection contains two modes of transmission, free-virus and synaptic transmission. (c) Kinetics of synaptic transmission. Synaptic transmission can be performed by means of different strategies that vary by *s*, the number of viruses transferred per synapse. If *s* is small, may synapses must be formed (sequentially in time). If *s* is large, the viral load is transmitted by means of few synapses.

We consider a modeling framework that builds on ordinary differential equations of virus dynamics [15], [16], [17], [18], [19], [20], and adds to modeling approaches that discussed cell-to-cell transmission in different contexts [19], [21]. Concepts are explained schematically in Figure 1 and the model is given as follows:
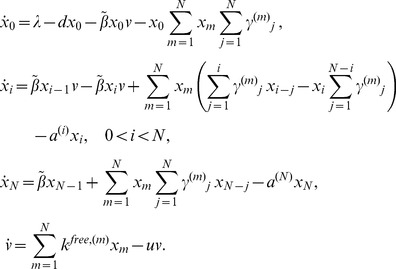
(1)


Here *x_0_* denotes uninfected cells, *v* denotes is the population of free virus. We assume that infected cells can be simultaneously infected by several copies of (genetically identical) viruses. The variable *x_i_* denotes the number of cells infected with *i* viruses, where the index *i* runs from *1* to *N*, the maximum number of viral genomes incorporated in a cell’s genome (that is, the maximum multiplicity of infection). Target cell production and death rates are given by *λ* and *d*. Infected cells die with a rate *a^(i)^*. It is assumed that a fraction of the viruses produced by a cell is transmitted via the free-virus pathway. The remaining fraction is transmitted via the synaptic pathway. The free-virus pathway is represented by terms multiplying parameter 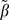. For this pathway, virus is produced by infected cells at the rate 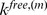, which can be a function of the cell’s multiplicity of infection. Free virus decays with rate *u*. The cell-cell transmission pathway is represented by terms multiplying 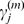, which is the probability for a cell with multiplicity of infection *m* to successfully transmit *j* copies per synapse. For convenience, all the parameters are summarized in Table 1. Note that both pathways lead to the transmission of whole virions and that in this respect the transmitted entity is biologically the same.

**Table 1:** Model parameters and their definitions.

Infection dynamics parameters	
*λ*	Production term of the target cells
*d*	Death rate of uninfected cells
*a*	Death rate of infected cells
*β ^syn^*, *β ^free^*	Infectivity of the synaptic and free-virus pathways

In our model we used the superscripts in parenthesis to indicate the explicit dependence of certain parameters on the multiplicity of infection. For example, in general, the parameter 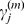 depends on the number of resident viruses in the infected cell. If however we assume that the role of the multiplicity of infection is negligible, system (1) collapses to a very simple two-component model,
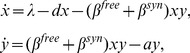
(2)where × denotes the number of uninfected cells, y the total number of infected cells, the number of free viruses is assumed to be in quasi-steady state [16], and the rates of infection for the two pathways are given by 
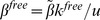
 and 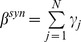. Thus the full system (1) is an equivalent of simple system (2), where we include the dependence of various processes on the multiplicity of infection.

Both systems (1) and (2) represent the overall process of infection, see figure 1(a). The two infectivity parameters, 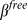 and 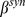, reflect the two transmission pathways, free-virus and cell-to-cell, see figure 1(b). In the next section, we go beyond this level of description and connect the virus dynamics equations with the kinetics of virus production and transmission in the context of the synaptic transmission pathway, figure 1(c).

#### Kinetics of synaptic transmission

**Figure 2:**
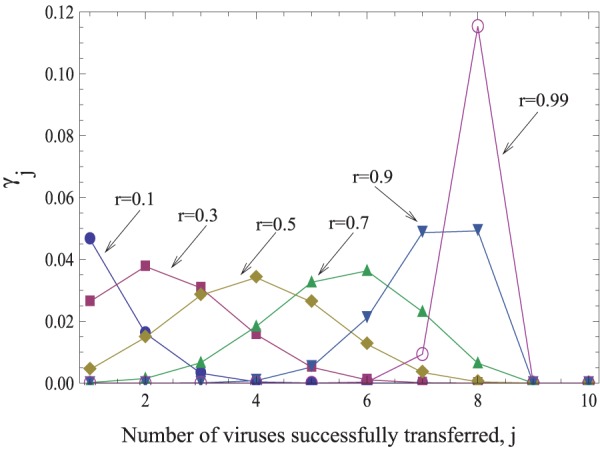
The functions γ_j_, the probability to successfully transmit *j* viruses, given strategy *s = 8*, for different values of the infectivity parameter, *r*. We have 
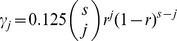
.

In general, we define a viral synaptic strategy as a probability distribution, *{q_i_^(m)^}*, which reflects the probability for an infected cell of multiplicity *m* to attempt to transfer *i* viral particles per synapse. The rate of synaptic viral transfer is given by 
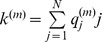
, and the rate of synapse formation is 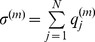. We denote by *s* the mean number of viral particles that a source cell attempts to transmit to its target. The parameter 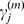 (the probability for a cell of multiplicity *m* to successfully transfer *j* viruses to a target cell per synapse) depends on the cell’s strategy and also on the probability for an individual transferred virus particle to survive and successfully infect a target cell, which is denoted by *r*. An example of the probability distribution 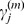 for a fixed strategy, a fixed multiplicity *m*, and for different values of *r* is given in figure 2. There, we made the simplifying assumption that an infected cell either attempts to transfer *s* viruses to the target cells with probability 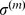, or it transfers *0* viruses with probability 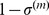. In this case, the value *s* completely characterizes the strategy. Therefore, in the rest of the paper we will simply refer to different strategies by the corresponding number, *s*. Using this simplification allows us to gain analytical insights when examining the costs and benefits of the different transmission strategies. In the Supporting Information S1, it is shown that conclusions remain the same for the more realistic case, where the number of transferred viruses is drawn from a probability distribution with a characteristic average. Note that in this model, synaptic transmission from the source cell to the target cell leads to multiple infection by genetically identical viruses. Simultaneous infection with different virus strain is explored below in the context of evolutionary dynamics.

Using these modeling approaches, we can relate the important parameters of infection dynamics, such as the infectivity 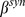 or the basic reproductive ratio 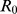 of the virus, with the viral strategy *s*. Our main goal is to explore how changing the number of viruses transferred per synapse (strategy *s*) affects the viral fitness measured by 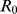.

### The Base-line Scenario

**Figure 3:**
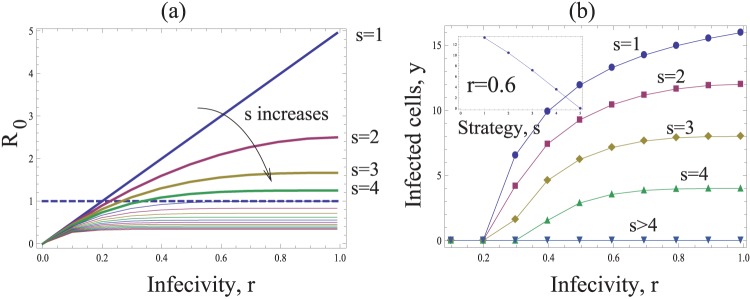
The basic model of virus dynamics with synapses. The basic reproductive ratio *R_0_* (a) and the total number if infected cells *y* (b), are plotted as functions of the infectivity *r*. The horizontal dashed line in (a) corresponds to the infection threshold, *R_0_ = 1*. The strategies capable of establishing successful infection for the given parameters are plotted by thick lines in (a). The inset in (b) plots the number of infected cells as a function of strategy, *s*, for a fixed value *r = 0.6*. Other parameters are: *N = 15*, λ* = 200*, *d = 4*, *a = 10*.

We start by considering a simplified version of model (1) that only takes into account synaptic transmission. We examine the effect of varying the number of viruses transferred per synapse (the strategy) on the basic reproductive ratio of the virus under different conditions. Subsequently, the full model is used to examine the synaptic transmission strategies in the context of concurrent free-virus transmission. Our base-line model makes the following assumptions:

1. The burst size of infected cells and their death rate are the same regardless of the number of resident viruses in the cell, *m*. Thus, the mean number of virus particles that a cell produces and attempts to transmit does not depend on the multiplicity, *m*: 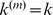. We further assume that the death rate of infected cells is independent of the multiplicity of infection: 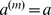.
2. The total number of viruses that a cell will transmit via synapses during its life-span is independent of the strategy *s*. Thus, if *s* is small, this means that a cell attempts to pass a small number of particles to many cells by forming many synapses. If *s* is large, then the cell’s strategy is to transfer many viral particles to a few cells, by forming few synapses, figure 1(c). Mathematically, we will assume the following relationship between the rate of synapse formation and the intensity of virus production destined for synaptic transmission:

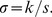
(3)Under these assumptions, we define the basic reproductive ratio of the virus, *R_0_*. It denotes the average number of newly infected cells generated by a single infected cell at the beginning of the infection, and needs to be greater than one for the virus population to become established [16]. The basic reproductive ratio of the virus also tends to correlate with viral fitness in virus dynamics models [16], which is shown below to be also true for our model. In this system it is given by 
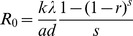
 (Supporting Information S1). Figure 3(a) shows *R_0_* as a function of the probability *r* that individual viruses successfully infect a cell following entry. Different curves are shown for different values of *s* (i.e. the number of viruses transferred per synapse). As expected, *R_0_* increases for higher values of *r*. However, an increase in the number of transferred viruses, *s*, leads to a decline in the basic reproductive ratio of the virus, which is also obvious from the expression for *R_0_*. If the value of *s* lies above a threshold, the infection cannot be maintained anymore. Therefore, the most efficient strategy is the transfer of a single virus particle per synapse (*s = 1*), while the least efficient strategy maximizes the number of transferred viruses per synapse. Similar trends are shown when considering the equilibrium number of infected cells (figure 3(b)). Higher numbers of transferred viruses, *s*, lead to lower numbers of infected cells.

This result can be understood intuitively. Suppose that *r* is relatively high, such that a virus transferred by an infected cell has a high chance of infecting a target cell. If many viruses are transferred to a cell, leading to multiple infection, this collection of viruses still gives rise to a single infected cell. If they were distributed among different cells, the same collection of viruses would give rise to more infected cells. Multiple infection through synaptic spread essentially “wastes” viruses that could otherwise infect other cells. The same argument holds for lower values of *r*, but the differences among strategies are less pronounced then.

The following sections will examine conditions under which the basic reproductive ratio of the virus can be maximized for larger number of viruses transferred per synapse. The exact number transferred viruses that maximize *R_0_* in these models depend on unknown parameters and figures are meant to illustrate general model behavior rather than predictions based on measured parameters. The demonstrated model behavior works for a very large range of parameters, as shown in the Supporting Information S1.

### Infected Cell Burst Size and the Multiplicity of Infection

In the basic model we assumed that the burst size of infected cells was independent of the multiplicity of infection. Here, we explore the assumption that cells with a higher number of resident viruses tend to produce and transfer more virus particles. More precisely, the parameter *k* now depends on *m*, such that 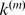 is a growing and possibly saturating function of multiplicity *m*. As an example, we consider the function 
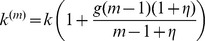
. The case 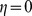 corresponds to the old assumption where the multiplicity of infection does not make a difference. The case 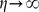 corresponds to the unsaturated growth of the virus production with the number of resident viruses. Finite values of 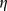 produce saturated growth of the virus production.

**Figure 4:**
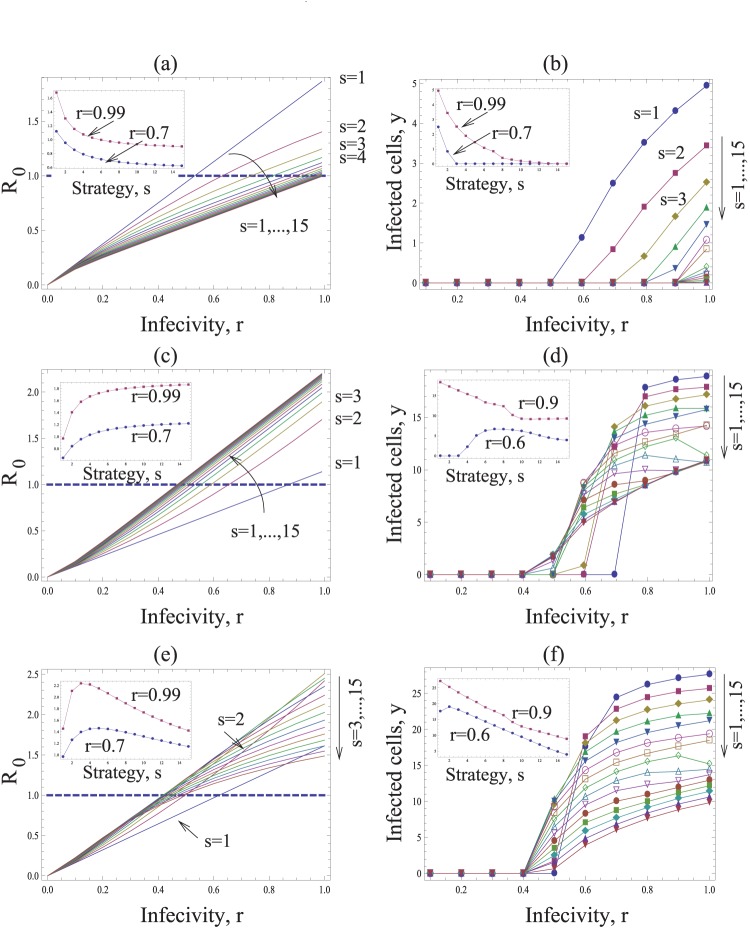
The dependence on the multiplicity of infection. Same as in figure 3, except the rate of virus production is given by 
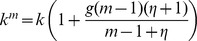
 (a,b) Subadditive dependence without saturation (adding saturation does not change the picture qualitatively), *g = 1/2*, *λ = 8*, *d = 0.5*, 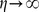. (c,d) Superadditive dependence without saturation, *g = 2*, *λ* = 20, *d = 0.6*, 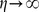. (d,e) Superadditive dependence with saturation, *g = 2*, *η = 10*, *λ* = 30, *d = 0.6*. The insets in (a,c,e) plot the basic reproductive ratio as a function of strategy, *s*, for two fixed values of *r = 0.9*. The insets in (b,d,f) plot the number of infected cells as a function of strategy, *s*, for a two fixed values of *r*. Other parameters are *a = 1*, *N = 15*, 
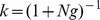
.

We observe the following patterns. Let us first assume that the effect of multiple infection on the burst-size is at most additive. That is, if the multiplicity of virus in the cell increases by a factor of A, the burst size increases by a factor A or less, which corresponds to the assumption *g<1* in our model. Then, as before, transferring fewer viruses (low *s*) is more efficient and leads to a higher basic reproductive ratio than the transfer of more viruses (high *s*), see Figure 4(a,b). This holds for all infection probabilities, *r*.

If however, coinfection is associated with a certain degree of cooperation of viruses, such that the effect becomes superadditive, then this picture may change. Superadditive means that a cell infected with e.g. two viruses has more than twice the burst size than a cell infected with a single virus (*g>1* in our model). Now, the basic reproductive ratio of the virus increases towards an asymptote with the number of viruses that are transferred per synapse, *s* (Figure 4c). This, however, assumes that the addition of viruses to a cell can lead to the same increase in the rate of virus production without bound. If we assume that the rate of virus production saturates as more viruses are added to the cell, then we find that an increase in the number of transferred viruses, *s*, first leads to an increase in *R_0_* towards a peak, followed by a decline as the parameter *s* is increased further (Figure 4e). Thus, the basic reproductive ratio of the virus is maximized for an intermediate number of viruses transferred per synapse. The exact value of *s* that optimizes *R_0_* is determined by the degree of saturation in the rate of virus production. If saturation occurs only at higher numbers of resident viruses, then the optimal value of *s* is higher.

These results can be shown by examining the basic reproductive ratio of the virus given by 
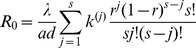
 (see Supporting Information S1 for details). For the unsaturated case, we have 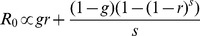, which is a decaying (growing) function of *s* for *g<1* (*g>1*). Therefore, in order for a higher value of *s* to maximize the basic reproductive ratio of the virus, the effect of coinfection must be superadditive.

The intuitive reason for this result is as follows. For an intermediate number of transferred viruses to optimize *R_0_*, the advantage gained by an increased burst size of coinfected cells must outweigh the cost incurred by the amount of “wasted virus”, hence leading to faster spread. This can be achieved if the burst size of multiply infected cells grows faster than the multiplicity of infection.

### The Effect of Immune Defense Saturation

**Figure 5:**
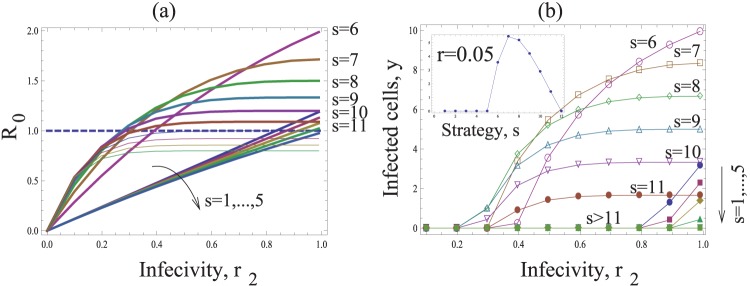
The effect of flooding the immune defense. Same as in figure 3, except for the function 
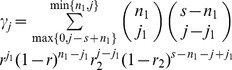
. The inset in (b) plots the number of infected cells as a function of strategy, *s*, for a fixed value *r = 0.05*. Other parameters are: *N = 1*, λ = 66.7, *d = 1.67*, *a = 3.33*, *k = 1*, *n_1_ = 5*, *r = 0.1r_2_*.

In this section, we will consider the effect of saturating cellular factors that can inhibit infection of the cell to a certain degree. It has been proposed that the viruses entering cells are subject to inhibition by factors that can be considered part of innate immunity [22], [23]. The discussion section describes specific examples and their applicability to HIV infection. The individual factors bind virus particles with the effect of reducing their probability of successful infection. The resulting probability of infection is what we denoted by *r* in the previous sections. It is possible that the number of inhibiting factors that can bind the virus particles is limited, and thus a synapse that sends a large number of viruses into a specific target cell has a possibility to “flood” and saturate this defense [22]. Suppose that *n_1_* immune particles are available in the cell, then the first *n_1_* viruses will be bound to them, resulting in a low individual probability of infection per virus, *r*. If the number of viruses entering the cell by synapse, 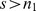, then the remaining 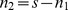 particles will have a higher probability of successfully infecting, 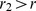. This leads to a different expression for the probability of successfully transmitting *j* viruses, 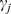. It then follows that an advantageous strategy is to transmit 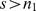 viruses, such that some of them will have a higher chance of infection. As a result, strategies which transfer an intermediate number of viruses per synapse maximize the basic reproductive ratio of the virus, as illustrated in figure 5.

**Figure 6:**
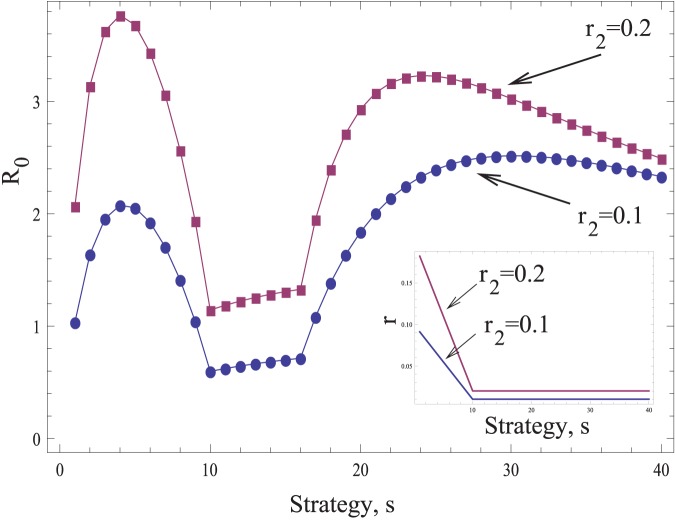
Virus-mediated induction of intracellular defense factors can lead to peaks in *R_0_* for two different viral strategies, *s*: a “stealth strategy” and a “saturation strategy”. Plotted is the basic reproductive ratio, *R_0_*, as a function of strategy, *s*, for two different values of the infectivity parameter, *r_2_*. The rest of the parameters are as follows: 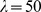, *d = 0.1*, *a = 4*, *n_1_ = 16*. The infectivity parameter *r* depends on the strategy. It is given by *r = r_2_-s(r_2_-r_2_/10)/10* if *s<10*, and *r = r_2_/10* if 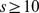. These functions are shown in the inset for the two values of *r_2_*.

The above analysis assumed that there is a fixed amount of immune effectors within the cell that are depleted in action. If intracellular immune effectors can be induced by the virus, however, we observe a slightly different pattern (Figure 6). This scenario assumes that the overall probability of infection, *r*, decays with an increasing number of viruses that are passed through the synapse, because more viruses induce more immune effectors (Figure 6, inset). When the number of transferred viruses, *s*, crosses a threshold, the immune effectors are induced maximally and no further reduction in the infection probability occurs. Now, the basic reproductive ratio of the virus, *R_0_*, peaks at two synaptic strategies, *s*. For low values of *s* the pattern is similar to the model without immune effectors, leading to peak *R_0_* for an intermediate value of *s* (*s = 4* in these simulations). This is a “stealth” strategy where the virus keeps a low profile and avoids strong degrees of inhibition. Transferring more viruses per synapse, *s*, leads to stronger induction of anti-viral effectors and to a reduction in *R_0_*. If the number of viruses transferred per synapse is increased even more, however, drug saturation of the virus is observed, which leads to a further rise in *R_0_* towards a second peak for high values of s. As before, even higher numbers of viruses transferred per synapse lead to wasting of viruses through infection of already infected cells and thus to a decrease in *R_0_*. Therefore, two “optimal” viral strategies are observed in this model: a stealth and a saturation strategy. Depending on the parameters, either could be more advantageous.

### Constraints during Synapse Formation

In the models above we assumed that in cell-cell transmission, all the virus particles produced were transferred via synapses. A more realistic assumption is that formation of each synapse takes a certain amount of time before the cells separate and are able to find new partners. The process of finding a partner might also be a rate-limiting step. In a spatial setting, cells have a limited number of neighbors, and therefore spread via the virological synapse is much more resource-limited than spread via free virus (the latter can occur over longer distances). Therefore, the rate of synapse formation may not be inversely proportional to the number of viruses transferred (the strategy), but rather may have a cap, that is, the maximum intensity of synapse transmission. In this model, for relatively low amounts of transferred viruses (low s), it is not possible to form enough synapses to transfer all the viruses produced. To incorporate this effect mathematically, instead of equation (3) we assume that 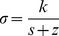, where *z* is a parameter. Case 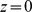 corresponds to the base-line model.

**Figure 7:**
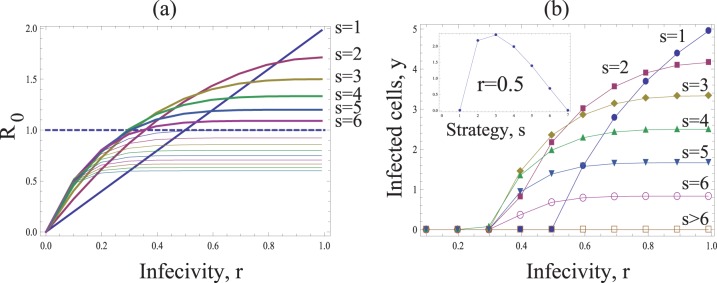
Limited ability of synapse formation. Same as in figure 3, except for the relationship 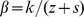. The inset in (b) plots the number of infected cells as a function of strategy, s, for a fixed value *r = 0.5*. Other parameters are: *N = 15*, λ* = 300*, *d = 5*, *a = 30*, *z = 5*, *k = 6*.

Figure 7 shows that under these assumptions, the strategies involving the transfer of few viruses (low *s*) may be less effective compared to strategies involving the transfer of more viruses per synapse (higher *s*). In general, for each given infection probability, *r*, there will be an intermediate optimal viral transfer strategy, *s*, which leads to the maximum value for *R_0_* and the maximum number of infected cells. The reason for this is directly related to the fact that synapses cannot be formed at arbitrarily high rates. Transfer of low numbers of viruses per synapse will end up wasting a lot of viruses because not enough synapses can be formed to transfer all the viruses produced.

This argument, however, assumes that viruses can only be transmitted through synapses. In reality, if a cell cannot form a sufficient number of synapses during its life-span to transfer all offspring virus, this remaining offspring virus population does not have to be wasted, but can be released as cell-free virus. Therefore, we re-consider this argument in the context of the full model (1) that takes into account both transmission pathways. Let us define the probability of successful infection by free virus as 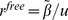. The result now depends on the magnitude of this parameter *r^free^*. If 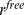 *<r*, the result is the same as before: for each given infection probability, *r*, there may be an intermediate optimal viral transfer strategy, *s*, which leads to the maximum value for *R_0_*. On the other hand, if 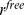 *>r*, the synaptic strategy with *s = 1* maximizes the basic reproductive ratio of the virus. In other words, the transfer of only one virus particle per synapse leads to the fastest rate of viral spread. Therefore, the relative infection probability characteristic of the two transmission pathways plays a deciding role. This can be influenced by the survival of the offspring virus. Thus, if offspring virus has a higher likelihood to be lost during free virus compared to synaptic transmission, then an intermediate number of viruses transferred per synapse maximizes *R_0_*. Note, however, that the exact nature of the condition can depend on the mathematical formulation of the rate limitations during synapse formation, and is explored in general terms in the Supporting Information S1.

### Synaptic versus Cell Free Transmission

So far, we have examined how different synaptic transmission strategies affect the basic reproductive ratio of the virus, and defined the optimal number of transferred viruses that maximize *R_0_* under different sets of assumptions. In a similar way, one can compare how synaptic and free virus transmissions contribute to the basic reproductive ratio of the virus. This can be addressed with the full model (1) taking into account both transmission pathways. The kinetic parameters of the infection process are likely different in these two modes of transmission. The viral production rate is given by the distinct parameter *k^free^* and the probability of successful infection per virus is given by 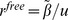. Therefore, from a theoretical perspective, free virus transmission can be more or less efficient than the optimal synaptic strategy, depending on the values of the parameters that describe the kinetics of free virus transmission versus synaptic transmission.

In general, the two routes of virus transmission described in system (1) are not independent, and have to be taken into account when studying the efficiency of different synaptic strategies. In this context, we described a model where during the life-span of the cell, the fraction of offspring virus transferred through synapses depends on the synaptic strategy, *s*. For low numbers of viruses transferred per synapse (low *s*), fewer synaptic connections are established during the life-span of a cell. Hence, a lower fraction of the total offspring virus produced by a cell is transferred through synapses, and a higher fraction is released into the extracellular environment. In this particular model, looking at both pathways is necessary (see Section 2 of Supporting Information S1 for full details of the model). In the other models considered, we found that including the free virus pathway does not change the outcome. For example, in the base-line model, the same number of viruses is passed through synapses during the life-span of the cell for any value of *s*. By varying *s*, we observe a difference in the overall viral replication rate of the virus coming from the synaptic pathway, 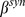. However, the component.


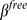
 coming from the free-virus transmission is not influenced by the strategy *s*. It remains an additive constant, which does not influence the result of the outcome of evolutionary competition. A similar argument works for the rest of the models we considered.

### Evolutionary Dynamics of Synaptic Strategies

In this paper we use the basic reproductive ratio, *R_0_*, as a measure of fitness of various synaptic strategies. Here we show that this quantity is indeed a valid measure of relative evolutionary advantage of competing traits. In order to do this, we formulate an evolutionary model where two virus strains characterized by different synaptic strategies (different values of *s*) compete for the same target cell population. Let us suppose that there are two competing strategies, *s_1_* and *s_2_*, and denote by *x_ij_* the number of cells infected by *i* copies of *s_1_*-virus and *j* copies of the *s_2_*-virus. We further denote by 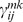
the probability that a cell infected by *m* copies of *s_1_*-virus and *k* copies of the *s_2_*-virus will successfully transfer *q* copies of *s_1_*-virus and *p* copies of the *s_2_*-virus by means of the synaptic transfer. Then, the dynamics of synaptic transmission is described by the system



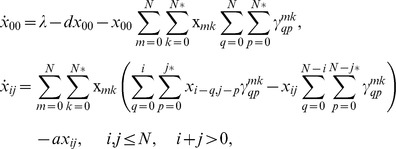
where the star in the upper limits of the double summations indicates the implicit assumption that the two indices cannot be zero simultaneously. This system is a direct generalization of system (1) for two competing strategies. For simplicity we assumed that the death rate is independent of the multiplicity of infection, and omitted free-virus transmission.

In order to analyze this system, we need to make further assumptions on the strategies of cells infected with two different types of virus. We considered several possible choices. Suppose that a cell is infected by *m* copies of *s_1_*-virus and *k* copies of the *s_2_*-virus. Then its strategy *s_mk_* can be given by (a) the mean strategy of the resident viruses, 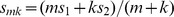, (b) the strategy of the majority of the resident viruses, (c) the strategy with the largest number of viruses transferred as long as there is at least one resident virus with this strategy. These assumptions only made a quantitative difference in the dynamics and did not affect the outcome of competition. We further assumed that a cell infected with both strains of viruses transfers them in proportion to their representation within the cell.

**Figure 8:**
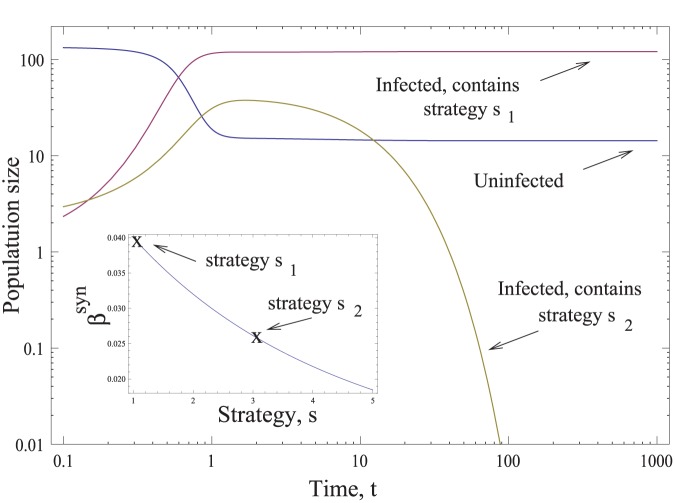
The evolutionary simulations. The time-dependent solution of the evolutionary virus dynamics simulation is presented (please note the log axes). The uninfected cell population is *x_00_*, and the infected populations are presented by two lines, one showing the sum of all cells containing the *s_1_* virus, 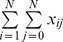, and the other containing the *s_2_* virus,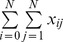. The inset shows the infectivity of the two strains. We used the base-line model for this simulation. The parameters are *s_1_ = 1, s_2_ = 3, z = 0, Q = 0.1,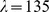, a = d = 1, N = 5.*

The result of a typical simulation is presented in Figure 8, where we ran the evolutionary system with two strategies, *s_1_ = 1* and *s_2_ = 3*, in the framework of the base-line model, where the first strategy has a larger basic reproductive ratio. For the parameters chosen, a successful infection is established, where only the viruses with strategy *s_1_ = 1* are represented. The population of cells containing *s_2_ = 3* viruses decays to zero. The competitive exclusion shown here has been observed for all the scenarios, which include models other than the base-line model. As long as a strategy possesses a higher basic reproductive ratio, it invades and drives the weaker strategy extinct. This supports the claim that the basic reproductive ratio is an appropriate viral parameter to characterize the fitness of the strategy in the context of synaptic transmission.

Finally, we would like to discuss the general evolutionary approach we pursue in this paper and put it in a greater context. In our line of thinking we implicitly assume that if a feature is present then it must be adaptive [24]. Or, on a similar note, if a virus possesses the largest fitness it will evolve. This “adaptationist” approach is explained and criticized in a paper by Gould and Lewontin [25]: “The immediate utility of an organic structure often says nothing at all about the reason for its being”. It is absolutely true that some traits of organisms may be present for reasons other than evolutionary advantage: they could be there as a consequence of multiple constraints that evolution itself imposes. These constraints may for example be related to the “history” of evolutionary change, the so-called “phyletic” constraints, or they could be a consequence of the “physics” of the organism's architecture. For example, viruses might be constrained to a subset of strategies and cannot achieve optimal infectivity. Gould and Lewontin write that these “constraints restrict possible paths and modes of change so strongly that the constraints themselves become much the most interesting aspect of evolution”.

It is not possible to argue positively whether the optimal solution found in our models can be reached, due to evolutionary time constrains or other constraints (which are not taken into account in our simple model). Therefore, the least we can do is provide an argument that relates the synaptic strategy with the infectivity and *R_0_* values of the virus, and say that, given enough time and no other constraints, this is the strategy that would evolve. This is the way in which we hope to contribute to the discussion of the evolutionary significance of different transmission strategies.

## Discussion

We constructed a mathematical model that describes the dynamics of viral infections, including two transmission pathways: cell-free transmission, and cell-to-cell transmission through viral synapses. This was motivated by the fact that besides free-virus transmission, synaptic spread has recently been suggested to play a major role in HIV infection, with source cells typically transferring tens to hundreds of viruses to the same target cell [4], [6]. This brought up the question of how synaptic transmission influences the rate of virus spread through the target cell population. Related to this is the question of how cell-cell transmission affects the fitness of the virus, measured by its basic reproductive ratio. In particular, we investigated different synaptic transmission strategies, defined by the number of viruses transferred per synapse. A number of scenarios were investigated. The most basic scenario gave rise to the prediction that the optimal viral strategy to maximize the rate of virus spread is the transfer of a single virus particle per synapse. Passing a larger number of viruses through synapses leads to the infection of already infected cells. This essentially wastes these viruses because they could be transmitted to uninfected cells instead, thus increasing the rate of viral spread. This result is interesting to consider in the context of a different viral infection. A recent study examined the *in vitro* growth and consequent plaque formation with vaccinia virus [26]. It was shown that newly infected cells expressed specific proteins that resulted in the “repulsion” of other viruses that attempted to infect the same cells. Thus, instead of coinfecting the cells, these viruses were “redirected” towards uninfected cells. Hence, vaccinia virus has evolved a mechanism to avoid multiple infection of cells, instead ensuring that more uninfected cells are being targeted. Experiments showed that this mechanism significantly accelerates the rate of virus growth in this system. This observation supports our theoretical notion that transferring many viruses to cells can be ineffective and disadvantageous because virus particles that could in principle enter uninfected cells are wasted by entering already infected cells. Although no viral synapses are formed in the vaccinia system, the spatial arrangement of cells during plaque formation has a similar effect in the sense that viruses released from a source cell are most likely to repeatedly reach the same set of target cells that are in their direct vicinity. The example of vaccinia shows that there is a certain selection pressure against transferring high numbers of virus particles to the same cell.

In the light of this, an explanation is required for the observation that on the order of 10^2^ virus particles are transferred through synapses in HIV infection [6], [13]. A number of scenarios were explored that could make an intermediate number of transferred viruses the optimal viral strategy, and these scenarios are discussed as follows.

A higher burst size of multiply infected cells could have this effect. While this can indeed elevate the efficiency of passing many viruses per synapse, the increase in burst size must be super-additive for this effect to be observed, e.g. doubly infected cells must produce and transfer more than twice as much virus as singly infected cells. There are currently no data that examine the burst size of infected cells in dependence of the infection multiplicity. A super-additive effect, however, is unlikely to occur unless special cooperative interactions between co-resident viruses occur. Cooperative effects have been observed in the context of unintegrated viral DNA, which could produce offspring virus in the presence of integrated virus rather than becoming a replicative dead end [27], although the contribution of this effect for the overall dynamics is currently unclear. Even if more viruses are produced in multiply infected cells, this could be canceled out by an increased death rate [11]. The effect of multiple infection on the kinetics of virus production and cell death remains to be determined.

Similarly, while saturation of intracellular defense factors can theoretically make it advantageous to pass many viruses per synapse, the relevance of this mechanism in HIV infection remains unclear. TRIM5α has been identified as an intracellular factor that inhibits HIV replication upon entry into the cell. It has been found to be especially effective at preventing HIV-1 infection in cells derived from Old World monkeys [28], [29], [30]. The human version of TRIM5α is, however, less protective against HIV-1. Members of the APOBEC family of restriction factors interfere with reverse transcription, although this effect is countered by viral Vif [23]. It is unlikely, however, that synaptic transmission can lead to the saturation of this factor because it is incorporated into the virion in the source cell before displaying activity during reverse transcription upon infection of the target cell. Similarly, factors such as tetherin probably are not applicable because viral assembly and budding is inhibited [23], and cannot be saturated by multiple infection. Nevertheless, experiments indicate that cells contain saturable targets that inhibit infection of cells [31] and that could be directly relevant to our model scenario, although they remain to be identified [22]. Saturation of anti-viral factors in target cells by multiple infection through virological synapses is being investigated increasingly, see [22] for a review.

Constraints during synapse formation can result in an optimal viral strategy where a larger number of viruses is passed per synapse. While it is conceivable that transfer of fewer viruses per synapse leads to the generation of more synapses and the infection of a larger number of cells, there is likely a limit to the number of synapses that can be formed by a source cell during its life-span, because formation of the synapse and the consequent viral transfer take up time, and because each infected cell has only a limited number of neighbors that it can realistically reach by synapse. Thus it is plausible to assume that especially for lower-*s* strategies, a further reduction in the number of transferred viruses does not lead to the infection of a higher number of cells. In this case, “saving” viruses for yet uninfected target cells would be a waste, and it would pay to transfer a larger batch. This argument only holds, however, if the infection probability of free virus lies below a threshold relative to the infection probability during synaptic transmission. Otherwise, the majority of viruses that fail to be transmitted through synapses would likely find target cells via the free virus pathway, and the best synaptic strategy would again be to transfer a single virus per synapse. It is currently unclear whether the life-span of free viruses is sufficiently short relative to that of synaptically transferred viruses. It is feasible that *in vivo*, the rate of virus loss in the extracellular environment significantly exceeds that occurring during synaptic transmission, even though a sizable amount of virus can be lost in the endocytic pathway during synaptic transmission [4]. Neutralizing antibodies can have a drastic impact on the survival of free virus. On the other hand, viruses passed through synapses could be less susceptible to antibody-mediated activity, although the effect of antibodies on synaptically transmitted viruses is currently controversial [4], [7], [32], [33], [34]. In addition to this uncertainty, the relationship between the number of viruses transferred per synapse and the number of synapses that can be formed during the life-span of the source cell is currently unknown. Formation of multiple simultaneous synapses could shift this relationship [35]. Detailed estimates of the relevant parameters that describe the kinetics of synaptic and free virus spread are required to obtain further insights.

While these are some biological mechanisms that could apply to HIV and render an intermediate number of viruses transferred per synapse advantageous, detailed investigation of the kinetics and measurement of relevant parameters is required to get further insights into whether a given hypothesis can be rejected or whether it is consistent with data. The mechanisms explored here do not take into account some important aspects of the viral evolution in vivo. It is possible that synaptic transmission leads to accelerated evolution of the virus to overcome certain selection pressures, most likely through the multiple infection of cells. Viral recombination is a well-known process that requires multiple infection [12]. However, synaptic transmission is likely to lead to the multiple infection of cells with identical viruses, even though mutation events upon infection can lead to the generation of a certain degree of diversity before integration. Another possibility is that the transfer of about *10^2^* viral particles per synapse does not represent the result of viral adaptation to maximize its fitness. It could be a side-effect of another process the benefit of which outweighs the cost associated with synaptic transmission. The important result in this paper is that in the most basic setting, the transfer of more than one virus per synapse leads to a reduction in the basic reproductive ratio of the virus, and thus to a lower fitness, despite synaptic transmission being very effective for virus transmission on a per cell basis. Hence, more work needs to be performed to account for its prevalent existence in HIV infection.

## Materials and Methods

The work described in this paper is based on ordinary differential equations, which have been explored analytically and numerically. Due to the extensive nature of this analysis, details are given in Supporting Information S1.

## Supporting Information

Supporting Information S1 This file provides mathematical details about the modeling approaches described in the main text.
(PDF)
