# Supplementary material for: Effect of Synaptic Transmission on Viral Fitness in HIV Infection
Source: PLoS One. 2012 Nov 15;7(11):e48361. doi: 10.1371/journal.pone.0048361 (PMC3499495; doi:10.1371/journal.pone.0048361)
Supplement: Supporting Information S1 — This file provides mathematical details about the modeling approaches described in the main text. (PDF) [file pone.0048361.s001.pdf]

Effect of synaptic transmission on viral fitness in HIV infection

Natalia L. Komarova, David N. Levy, and Dominik Wodarz

Supplementary Information

1 Cell-to-cell transmission strategies

Definition of strategies. Let us assume that infected cells have a given probability distribution to pass on a number of viruses, given by q_i, with $\sum_{i=0}^N q_i = 1$. The probability distribution, $\{q_i\}$, defines a strategy for cell-to-cell transmission.

Suppose that each virus has a probability to establish a successful infection, r, or fail with probability $1-r$. The probability to produce i viruses which successfully infect another cell is then

$$\gamma_j = \sum_{i=j}^N q_i \binom{i}{j} r^j (1-r)^{i-j}.$$

The mean number of viruses passed by the cell is

$$k = \sum_{i=1}^N q_i i. \quad (1)$$

The mean number of successful viruses produced by the cell is

$$V = \sum_{i=1}^N \sum_{j=i}^N q_j \binom{j}{i} r^i (1-r)^{j-i} i = \sum_{j=0}^N q_j \sum_{i=0}^j \binom{j}{i} r^i (1-r)^{j-i} i = \sum_{j=0}^N q_j j r = Qr,$$

where we interchanged the order of summation and then used the formula for the mean of the binomial distribution. The probability of successful infection is

$$\beta = 1 - \gamma_0 = 1 - \sum_{j=0}^N q_j (1-r)^j. \quad (2)$$

Strategies with a fixed transmission number. For simplicity we will consider the probability distributions of the form,

$$q_0 = 1-q, \quad q_s = q, \quad q_k = 0 \quad k \neq 0, \quad k \neq s.$$

In other words, a cell either passes s viruses or 0 viruses. For such a cell, we have

$$\gamma_j = q \binom{s}{j} r^j (1-r)^{s-j}, \quad 0 \le j \le s, \quad \gamma_j = 0, \quad j > s. \quad (3)$$

Further,

$$k = qs, \quad V = qsr, \quad \beta = (1-(1-r)^s)q.$$

For each strategy,

$$q = \frac{k}{s},$$

and therefore

$$R_0 = \frac{\lambda\beta}{ad} = \frac{\lambda k}{ad}\frac{1-(1-r)^s}{s}. \tag{4}$$

General cell-to-cell transmission strategies. A general strategy is defined by the vector $(q_1, \dots q_N)$ of probabilities to transmit a number of viruses. We will assume that all the strategies have an invariant, k, which is the mean number of viruses passed, see equation (1).

Consider the quantity β for a general strategy, equation (2). β is a growing function of r, with

$$\beta|_{r=0} = 0, \quad \beta|_{r=1} = 1 - q_0.$$

The last equality is obtained by taking the limit as $r \to 1$ of expression (2). Let us construct the quantity W, the expected number of viruses passed, conditioned on a successful transmission. We have

$$W = \frac{\sum_{m=1}^N q_m m}{\sum_{m=1}^N q_m} = \frac{k}{1-q_0}.$$

We can see that

$$\beta|_{r=1} = \frac{k}{W}.$$

In other words, the maximum probability of successful infection is inversely proportional to the conditional average of viruses transmitted. In the simple one-parametric family of strategies considered before, we have $W = s$.

Let us first consider the limit where r is very small. In this case we can expand the probability

$$\beta \approx kr + \frac{r^2}{2}(k - \langle s^2 \rangle),$$

where $\langle s^2 \rangle = \sum_{m=0}^N q_m m^2$. We can see that the higher the quantity $\langle s^2 \rangle$ for the strategy, the lower the rate of synapse formation, and thus the less efficient the strategy is. Since the value $\langle s \rangle \equiv k$ is fixed, the value $\langle s^2 \rangle$ can be expressed in terms of the variance of the distribution,

$$\langle s^2 \rangle = Var + k^2.$$

That is, we obtain the result that strategies with higher variances of virus transfer numbers will be less efficient.

Next we turn to the limit of large infection probabilities. We know that for one-parametric strategies, higher values of s correspond to lower efficiency. An interesting question is how this dependency is altered by changing the variance

of the distribution. To investigate this, we consider a specific distribution which is a two-parametric family:

$$q_0 = 1 - k/s, \quad q_{s+i} = \frac{k}{s(2n+1)}, \quad -n \leq i \leq n,$$

with all the other entries being zero. Here the parameter s defines the conditional mean of the distribution, and the parameter n the "width" of this distribution. We have $W = s$ for this distribution, and the variance is given by $k(s - k + \frac{n(n+1)}{3s})$. The rate of successful infection in this case is given by

$$\beta = \frac{k}{s}\left(1 - \frac{(1-r)^{s-n}(1-(1-r)^{2n+1})}{r(2n+1)}\right).$$

It can be shown that as before, β is a growing function of r and a decreasing function of s. Further, β decreases with n, which means that for a given s, strategies with wider distributions are less effective.

2 Limited rate of synapse formation

Suppose that in general, the rate of synapse production is given by $Qf(s)$, where Q is a constant and the function $f(s)$ satisfies the following properties:

- $df/ds < 0$,
- $f(s) \leq 1/s$,
- $\lim_{s\to\infty} f(s)s = 1$.

Then we have $k = Qf(s)s$. It follows that unless $f(s) = 1/s$, k is now an increasing function of s. A particular form of this function that we consider in detail is given by

$$f(s) = \frac{1}{s+z},$$

where the constant z measures to what extent synapse formation is a rate-limiting step. In this case we have

$$R_0 = \frac{\lambda k}{ad}\frac{(1-(1-r)^s)}{s+z}.$$

2.1 Interaction between free-virus transmission and the synaptic transmission

In the model presented above, for low values of s some viruses do not get a chance to be transferred by cell-cell transmission. It is possible that such viruses are "wasted". On the other hand, we can also assume that these viruses get transferred by the free-virus mechanism instead. Here we will explore this interaction

between the free-virus and cell-cell transmission pathways, where at low s the free-virus pathway takes over.

The rate of virus production in the context of cell-cell transmission is given by $k = \sigma s$. The rate of virus production in terms of free-virus infection is given by k^{free}, as it appears in system (1) of the main text. The total rate of virus production is given by

$$k^{tot} = k + k^{free} = Qf(s)s + k^{free},$$

and we assume that this rate is independent of s. This implies that the rate of free virus production is now s-dependent: $k^{free} = k^{tot} - Qf(s)s$.

We would like to compare the values of R_0 for different strategies. Since $R_0 = \frac{\beta\lambda}{ad}$, it is enough to compare the β-values. We have

$$\beta^{syn} = \sigma(1-(1-r)^s), \quad \beta^{free} = k^{free}\frac{\tilde{\beta}}{u} = r^{free}(k^{tot} - Qf(s)s),$$

where we introduced a short-hand notation, $r^{free} = \tilde{\beta}/u$. We would like to examine the quantity

$$\beta = \beta^{syn} + \beta^{free} = Qf(s)(1-(1-r)^s) + r^{free}(k^{tot} - Qf(s)s)$$

as a function of s for different values of other parameters. We can rewrite the above expression as

$$\beta = QF(s) + k^{tot}r^{free}, \tag{5}$$

where

$$F(s) = (1-(1-r)^s - r^{free}s)f(s).$$

We need to determine what strategy, s, maximizes the function $F(s)$ (and therefore the function β, and the basic reproductive ratio, R_0). We can write

$$\frac{dF}{ds} = -f'[-(1-r^{free}(s+f/f')) + (1-r)^s(1+\ln(1-r)f/f')] = 0, \tag{6}$$

assuming that $f'(s) \neq 0$. Solving this equation for s is equivalent to finding the intersections of two functions,

$$F_1(s) = e^{as}(1-r^{free}(s+f/f')), \quad F_2(s) = 1 - af/f',$$

where we defined $a = -\ln(1-r) \geq 0$. It is clear that the function F_1 grows exponentially as $s \to \infty$, while the function $F_2(s)$ grows linearly with s. Therefore, if we can show that for some value $s = j$, $F_1(j) < F_2(j)$, then we are guaranteed to have at least one root of equation (6) with $s > j$.

Let us impose an additional condition on the function $f(s)$. Suppose that the inequality

$$f(j) > -f'(j)j \tag{7}$$

holds for some values of $1 \leq j \leq j_1$. Then as long as

$$r^{free} < r_c^{free}(r) \equiv \frac{e^{-aj}(af(j) + (e^{aj}-1)f'(j))}{f(j) + jf'(j)}, \tag{8}$$

then equation (6) has a root for some value of $s > j$. For inequality (8) to be meaningful, its right hand side has to be possible, which is guaranteed as long as

$$af(j) > (e^{aj} - 1)|f'(j)|,$$

which imposes a condition on r. Note that under condition (7), inequality (8) always holds for low values of r, which can be seen by expanding inequality (8) in the Taylor series in terms of the small r.

To conclude, we have analyzed the possibility that synapse formation is a rate-limiting step, such that for low values of s, synapses cannot be formed fast enough to transfer the same total number of viruses as that in the case of high s. In this case, additional viruses are transferred via the free-virus transmission pathway. This phenomenon is captured by the function $f(s)$, which quantifies exactly the extent to which the number of viruses transferred by the cell-cell pathway is reduced for low values of s. We ask the question, in the framework of these assumptions, what is the most effective cell-cell strategy, s?

Our analysis shows that for a fairly general form of the function $f(s)$, an intermediate maximum of the function $\beta(s)$ is possible for small values of r, unless the relative efficiency of the free virus transmission is larger than a threshold (condition (8)). The function $\beta(s)$ includes contributions from both the synaptic transmission and the free virus transmission. If the free-virus transmission is characterized by relatively small losses, u, (or relative high probabilities of individual virus infection, $\tilde{\beta}$), then the most efficient cell-cell strategy corresponds to $s = 1$. In this case, relatively more viruses will be transferred via the free-virus pathway, which is more efficient than the cell-cell pathway. On the other hand, if the free-virus pathways is less efficient (condition (8) for r^{free}), then the best strategy corresponds to intermediate values of s. For high values of r, the $s = 1$ strategy may be the most advantageous again, because in this case, higher-s strategies are too "wasteful", and are strongly disadvantageous.

2.2 A specific case

Let us next assume that

$$f(s) = \frac{1}{s+z},$$

where the value z measures the extent to which synapse formation is a rate-limiting step. In particular, $z = 0$ corresponds to the simpler model considered above.

In this case, the function $F(s)$ in equation (5) is given by

$$F(s) = \frac{1 - (1-r)^s - r^{free}s}{s+z},$$

and the equation $dF/ds = 0$ is equivalent to the equation $F_1 = F_2$, with

$$F_1(s) = e^{as}(1 + r^{free}z), \quad F_2(s) = 1 + a(s_z).$$

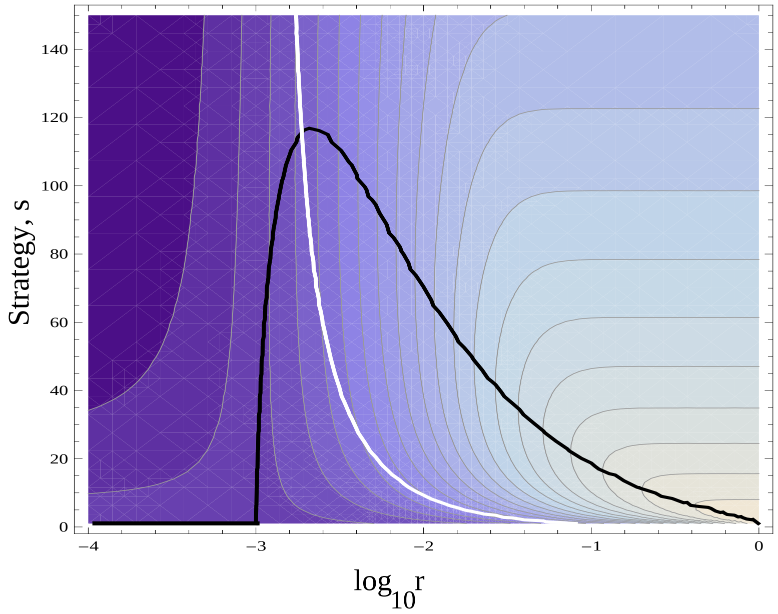

Figure 1: The contour-plot of the total infectivity β as a function of s and r. The lighter shade corresponds to higher values of β. The thick black line shows the location of the maxima of β, and the white line corresponds to the equation $\beta^{syn} = \beta^{free}$; in this plot, below the while line we have $\beta^{free} >> \beta^{syn}$, and the inequality is reversed above the while line. The parameters are: $r^{free} = 10^{-3}$, $z = 40$, $Q = 0.5$, $k^{tot} = 1$.

For this particular choice of the function $f(s)$ the analysis is very simple, as $F_1(s)$ is an exponential function and $F_2(s)$ is a linear function. We observe that if $z = 0$, the two functions $F_1(s)$ and $F_2(s)$ intersect once at $s = 0$, which means that for all $s \geq 1$, $dF/ds < 0$, and the most effective strategy is $s = 1$. For $z > 0$, the function $F(s)$ may have an intermediate maximum. This happens for a value of s with $s > j$ if

$$r^{free} < r_c^{free}(j) = \frac{e^{-aj}}{z}(1 - e^{aj} + a(j + z)). \quad (9)$$

Here by $r_c^{free}(j)$ we denote the threshold value of r^{free} such that below this value, one may have an intermediate maximum with $s > j$, that is, a strategy with with $s > j$ is the most effective one. In figure 1 we show the contour-plot of the infectivity $\beta = \beta^{free} + \beta^{syn}$ as a function of the synaptic strategy s and probability of infection r, for a fixed value of $r^{free} = 10^{-3}$ (for other parameter values, see figure caption). The thick black line shows the maximum value of β, and the white line corresponds to the equation $\beta^{syn} = \beta^{free}$. We can see that for larger values of r, the maximum of β corresponds to intermediate values of s. For lower values of r, the strategy $s = 1$ is the most efficient one. For this

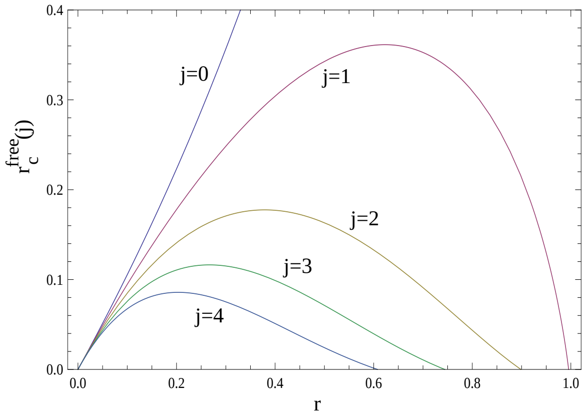

Figure 2: The threshold values of r^{free}, $r_c^{free}(j)$, plotted as functions of r for several values of j. If $r^{free} < r_c^{free}(j)$, this means that the most efficient cell-cell strategy corresponds to a value of s larger than j. Parameters are as in figure 1.

choice of function $f(s) = 1/(s+z)$, equation (8) is given by equation (9), which for $j = 0$ becomes

$$r_c^{free}(0) = a = -\log(1-r) \approx r.$$

In other words, for $r^{free} < r$, the most efficient strategy corresponds to $s = 1$. This is what we observe in figure 1.

Figure 2 plots of the threshold values of $q_c(j)$ as functions of r. We can see that $r_c^{free}(j)$ decay with j, that is, having a higher value for the most efficient strategy requires the free-virus strategy to be less efficient. Also, we can see that for high values of r, $r_c^{free}(j)$ corresponding to $j > 1$ attain negative values, which means that in that region, the strategy $s = 1$ is the most efficient one, see figure 1.

3 Multiplicity-dependent strategies

Suppose that the probability distribution, $q_i^{(m)}$, depends on the index m, the number of viruses in the transmitting cell, by assuming that the total number of viruses transferred is a function of the cell's multiplicity of infection: $k^{(m)}$. We assume that $k^{(m)}$ is a non-decreasing function of m. Let us consider the simplest choice of strategies, such as

$$q_i^{(m)} = \begin{cases} 1 - k^{(m)}/s, & i = 0, \\ k^{(m)}/s, & i = s, \\ 0, & \text{otherwise.} \end{cases}$$

3.1 The bifurcation analysis

We investigate the bifurcation of the no-infection solution of system (1) of the main text,

$$x_0 = \frac{\lambda}{d}, \quad x_i = 0, \quad 1 \leq i \leq N. \tag{10}$$

Let us consider r to be the control parameter. As the infectivity grows, infection gets established. We want to find the value $r = r_c$ for which solution (10) loses stability. This corresponds to an eigenvalue of the Jacobian, J, evaluated at solution (10) having a zero real part. Let us denote by I the unit matrix of size $N \times N$. We have

$$J = -aI + \begin{pmatrix} a-d & -\frac{\lambda}{d}\sum_{k=1}^{N}\gamma_k^{(1)} & -\frac{\lambda}{d}\sum_{k=1}^{N}\gamma_k^{(2)} & \dots & -\frac{\lambda}{d}\sum_{k=1}^{(N)}\gamma_k^{(N)} \\ 0 & \frac{\lambda}{d}\gamma_1^{(1)} & \frac{\lambda}{d}\gamma_1^{(2)} & \dots & \frac{\lambda}{d}\gamma_1^{(N)} \\ 0 & \frac{\lambda}{d}\gamma_2^{(1)} & \frac{\lambda}{d}\gamma_2^{(2)} & \dots & \frac{\lambda}{d}\gamma_2^{(N)} \\ \dots & & & & \\ 0 & \frac{\lambda}{d}\gamma_s^{(1)} & \frac{\lambda}{d}\gamma_s^{(2)} & \dots & \frac{\lambda}{d}\gamma_s^{(N)} \\ 0 & \dots & & & 0 \\ \dots & & & & \\ 0 & \dots & & & 0 \end{pmatrix} \tag{11}$$

The first eigenvalue of this matrix is equal to $d \neq 0$. We also have the eigenvalue a of multiplicity $N - s$. To find the rest of the eigenvalues, consider the $s \times s$ matrix $\tilde{J} = \{m_{ij}\}$, whose entries are given by

$$m_{ij} = \frac{\lambda}{d}\gamma_i^{(j)} = \frac{\lambda}{d} r^i (1-r)^{s-i} \frac{k^{(j)} s!}{si!(s-i)!} \equiv \mu_i \nu_j.$$

The remaining eigenvalues of the matrix J are related to eigenvalues of the matrix $\tilde{J}$ by $\Lambda - a$, where Λ is an eigenvalue of $\tilde{J}$. Matrix $\tilde{J}$ has the eigenvalue 0 with multiplicity $s - 1$, which corresponds another $s - 1$ eigenvalues of size a for matrix J. Finally, we have $\Lambda = \sum_{i=1}^{s} \mu_i \nu_i$. Therefore, the expression for the last eigenvalue of J is

$$\frac{\lambda}{d}\sum_{i=1}^{s} r^i (1-r)^{s-i} \frac{k^{(i)} s!}{si!(s-i)!} - a.$$

Equating this expression to zero we obtain the equation for the bifurcation threshold parameter, or the basic reproductive number of this system,

$$R_0 = \frac{\lambda}{ad}\sum_{i=1}^{s} r^i (1-r)^{s-i} \frac{k^{(i)} s!}{si!(s-i)!}. \tag{12}$$

The virus-free equilibrium is unstable if $R_0 > 1$. The equation $R_0 = 1$ gives us the threshold value r_c, such that for $r > r_c$ solution (10) loses stability.

If $k^{(m)} = k$, we obtain

$$R_0 = \frac{\lambda k}{ad} \sum_{i=1}^{s} \frac{r^i(1-r)^{s-i}}{s} \frac{s!}{i!(s-i)!} = \frac{\lambda k}{ad} \frac{1-(1-r)^s}{s}, \tag{13}$$

see expression (4). This is a growing function of r which is equal to r for $s = 1$, and is a saturating function for $s > 1$, with the value of $1/s$ at $r = 1$. As s grows, this function decreases. Therefore, if $k^{(s)} = 1$, the intersection of this function with the constant function 1 shifts to the right. This means that for constant $k^{(m)}$, r_c is a growing function of s. It is harder for larger s to establish a successful infection.

This trend can potentially be reversed if $k^{(m)}$ grows with m. Consider the class of functions $k^{(m)}$,

$$k^{(m)} = k \left(1 + \frac{g(m-1)(1+\eta)}{m-1+\eta}\right). \tag{14}$$

The parameter g tells us how quickly the rate of virus transmission increases with the multiplicity of infection of the cell, m, and parameter η is responsible for the saturation of this function for high values of m. The simplest case when $\eta \to \infty$ corresponds to the absence of saturation,

$$k^{(m)} = k(1 + g(m-1)). \tag{15}$$

The value $g = 0$ corresponds to the model of constant k. The regime with $g < 1$ corresponds to subadditive effect of coinfection. This is because for $g < 1$, we have $k^{(m_1+m_2)} < k^{(m_1)} + k^{(m_2)}$. This inequality is reversed if we have $g > 1$, which corresponds to superadditive, or cooperative, behavior of coinfection. The special case $g = 1$ describes an additive effect of coinfection. We can calculate the parameter R_0 in the case where the rate of virus transmission size is given by formula (15):

$$R_0 = \frac{\lambda k}{ad} \left(gr + \frac{(1-g)(1-(1-r)^s)}{s}\right).$$

We can see that $g = 0$ corresponds to the constant-k formula, (13). The case $g = 1$ gives $R_0 = \lambda kr/(ad)$, that is, the basic reproductive ratio is independent of the strategy, s, and is proportional to the probability of viruses to infect a cell. For any $0 \leq g < 1$, the function R_0 is a decaying function of s, because the function $\frac{1-(1-r)^s}{s}$ is a decaying function of s. Finally, for all $g > 1$, R_0 grows with s, and saturates at a constant level, $\lim_{s\to\infty} R_0 = gr$. In other words, starting from a certain values of s, all high-s strategies are more or less equally effective.
